# Strategies for hemodynamic maintenance of potential brain-dead donor: integrative review

**DOI:** 10.31744/einstein_journal/2021RW5630

**Published:** 2021-06-17

**Authors:** Beatriz Sousa da Fonseca, Verusca Soares de Souza, Taynara Oliveira Farias Batista, Guilherme Malaquias Silva, Dandara Novakowski Spigolon, Neide Derenzo, Aline Barbieri

**Affiliations:** 1 Universidade Estadual do Paraná ParanavaíPR Brazil Universidade Estadual do Paraná, Paranavaí, PR, Brazil.; 2 Universidade Federal de Mato Grosso do Sul CoximMS Brazil Universidade Federal de Mato Grosso do Sul, Coxim, MS, Brazil.

**Keywords:** Brain death, Hemodynamics, Nursing care, Organ transplantation

## Abstract

**Objective:**

To learn about the scientific production on strategies adopted for hemodynamic maintenance of brain-dead patients.

**Methods:**

Integrative review with articles published between 2007 and 2019, in Scientific Electronic Library Online (SciELO), Latin American and Caribbean Health Sciences Literature (LILACS), PubMed^®^ and ScienceDirect. The descriptors “ *Hemodinâmica* AND *Morte Encefálica* ” and “Hemodynamics AND Brain Death” were used. Exclusion criteria were non-human research and gray literature.

**Results:**

A total of 21 articles were listed. As strategies, the use of drugs – noradrenaline (n=8), vasopressin (n=7), dobutamine (n=6), hydrocortisone (n=4) and methylprednisolone (n=4); invasive (n=10) and noninvasive (n=13) cardiac monitoring; control of ventilatory parameters (n=12); and correction of fluid and electrolyte disturbances (n=17) were highlighted.

**Conclusion:**

The main strategies found in this integrative review were regulation of blood pressure and temperature, use of catecholamines and corticosteroids, in addition to the need for an early diagnosis of brain death. However, the lack of clearer protocols on the subject is notorious, making management with the potential donor difficult.

## INTRODUCTION

Organ transplantation is the most effective therapeutic alternative for many patients with end-stage diseases, and the waiting list for transplants has increased year after year. Ethical conflicts and family perception have been studied, noting a gradual increase in research related to this topic since 2010, demonstrating the need for a thoughtful analysis so that the organ donation process can be elucidated.^( 1 )^

Therefore, health education is reinforced as a relevant aspect for the dissemination of information on organ donation and brain death (BD), which highlights the importance of the participation of the multidisciplinary health team.^( 2 )^ But the lack of more comprehensive evidence on the subject denotes the need for clearer guidelines to provide uniform management of individuals requiring this care. Hence, it is important to consider the clinical and bioethical aspects.^( 3 )^

Brazil ranks second in number of transplants, behind the United States.^( 4 )^There was an increase of only 2.4% in effective donors in 2018, from 16.6 per million population (pmp), in 2017, to 17.0 pmp, in 2018. This rise was due to higher notification rate of potential donors by 2.2% of the donation attainment rate. The family refusal rate remained at 43%, lower in Paraná (27%) and higher in Mato Grosso (80%). In 2019, the number of effective donors was 18.1 pmp, with 40% of family refusal.^( 4 )^

In spite of this, the lack of Brazilian research on the subject is notorious. The systematized and detailed production and publication of information would contribute to the occurrence of transplants, and would help in the process of diagnosing BD, in the protection of potential donors, and with subsidies in education for professionals and family members.^( 5 )^

BD is characterized by the complete and irreversible loss of brain functions, defined by the cessation of cortical and brainstem activities.^( 6 , 7 )^In its protocol, the *Conselho Federal de Medicina* (CFM), established by Resolution 2,173 of 2017, provides that to undergo diagnostic procedures to determine BD, the patient must present all of the following requirements: presence of brain damage of known and irreversible cause; absence of treatable factors that would hinder the diagnosis; treatment and observation at hospital for a minimum of 6 hours; body temperature above 35^o^C, and arterial saturation according to criteria established by the Resolution.^( 6 )^

Brain death and organ and tissue transplantation are linked, since with BD patients can become potential multiple organ donors, which depends on their general condition.^( 8 )^ Thus, ensuring the functional maintenance of organ systems in the period between diagnosis and family interview is the main objective of the health team, to prevent organ impairment and making donation impossible.

The Intra-Hospital Organ and Tissue Donation for Transplant Committee (CIHDOTT) and the Organ Procurement Organization (ORO) are responsible for communicating the death to the State Transplant Center and for supporting the intensive care professionals in maintaining the potential donors.^( 1 )^ Thus, the importance of interdisciplinarity and teamwork is emphasized, so that the process is effective in the managing the pathophysiological repercussions of BD, hemodynamic monitoring, and provision of individualized care.

Having determined the importance of hemodynamic control in the maintenance of the potential organ donor in the occurrence of BD, the following question is posed: What are the strategies frequently adopted for hemodynamic maintenance in BD patients?

## OBJECTIVE

To learn about the scientific production on strategies adopted for the hemodynamic maintenance of potential brain-dead donors.

## METHODS

This is an integrative review, developed according to the following steps: identification of the theme and preparation of the research question; establishment of inclusion and exclusion criteria; search and categorization of studies; evaluation of included articles; interpretation of results; synthesis of knowledge; and presentation of the review.^( 9 )^

The search was conducted from November 2018 to February 2019, driven by the question: What are the strategies often adopted for hemodynamic maintenance in BD patients? The electronic databases chosen for searching articles were the Latin American and Caribbean Health Sciences Literature (LILACS), PubMed^®^, Scientific Electronic Library Online (SciELO), and ScienceDirect. The descriptors used were “ *Hemodinâmica* AND *Morte Encefálica* , from the Health Sciences Descriptors (DeCS), and “Hemodynamics AND Brain Death,” from the Medical Subject Headings (MeSH).

**Figure 1:**
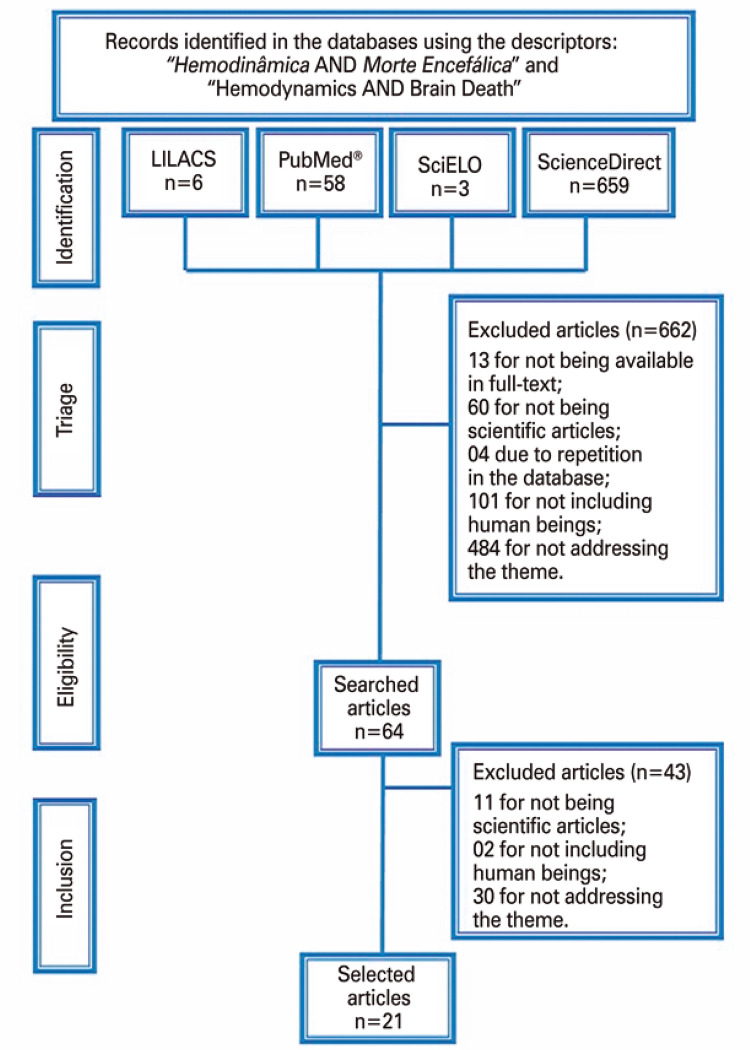
Literature search flowchart

LILACS: Latin American and Caribbean Health Sciences Literature; SciELO: Scientific Electronic Library Online.

Articles made available by universities or free of charge, and in full text were included; in Portuguese, English, or Spanish, and published between 2007 and 2019. Studies that were not conducted with humans, were not scientific articles, and were repeated in the databases were excluded. The selection flow is described in figure 1 .

For analysis of the articles, we used our own instrument that extracted information related to authors, journal, year of publication, Impact Factor, objective, and conclusion. This information was presented on a summary table.

In a complementary way, a checklist was drawn up, organized in dichotomous answers (yes and no), based on the hemodynamic maintenance form made available to the CIHDOTT of Paraná, which includes some care procedures with BD patients: absence of sedation, prevention of hypothermia, correction of fluid and electrolyte disturbances, start/maintenance of enteral diet, eye protection, noninvasive or invasive cardiac monitoring, glycemic control, transesophageal echocardiography, control of ventilatory parameters, control of central venous pressure, control of level of consciousness, and protective mechanical ventilation. Furthermore, all items indicated by the articles as hemodynamic maintenance actions for the potential donor were included in the collection, even if not taken into consideration by the instrument initially prepared, such as medications and their respective dose indication.

The articles were listed in order of selection and, for the indicated care, the analysis of frequency and percentage was performed.

## RESULTS

A summary of the 21 articles that make up this study, plus their country of origin and the journal’s Impact Factor, is presented on table 1 . Of the 21 articles listed, 95.2% (n=20) were published in foreign journals and only one in a Brazilian journal. Among the selected foreign articles, 23.8% (n=5) were from the United States; 14.2% (n=3) from France; and 9.5% (n=2) from Italy. Uruguay, Romania, Iran, United Kingdom, Turkey, South Korea, Belgium, Sweden, Lithuania, and the Netherlands totaled up 42.8%, with each country having one article.

**Table 1:** Summary of articles on hemodynamic maintenance in brain death

Author	Country	Impact factor	Objective	Conclusion
Plurad et al.^(10)^	United States	2.403	To determine the effect of arginine vasopressin on organ recovery rates	The use of arginine vasopressin with hormone replacement therapy is associated with increased organ recovery rates. This strategy should be universally adopted in donor maintenance
Dhar et al.^(11)^	United States	2.872	To compare a lower-dose steroid protocol (suitable for stabilization of adrenal insufficiency and sepsis) to the traditional high-dose regimen in maintaining organ BD donors	A lower dose corticosteroid protocol did not result in worsening donor lung or cardiac function compared to the traditional high dose regimen. Insulin requirements and glycemic control were improved. High-dose methylprednisolone may not be necessary to support BD donors
WestphaI et al.^(12)^	Brazil	0.806	To establish guidelines for the care and maintenance of adult organ donors, guiding and standardizing the care of BD potential donors	A protocol was developed with actions considered essential for organ donor maintenance: body temperature limits, laboratory tests indicated, blood pressure limits, and catecholamines used
Godino et al.^(13)^	Uruguay	0.806	To identify and quantify the causes of exclusion of potential heart donors, and define the risk factors for ventricular dysfunction among the BD population	Hemodynamic dysfunction in young people with head trauma was the main cause of heart donor loss. The use of vasopressors was observed in all patients investigated
Birtan et al.^(14)^	Turkey	0.806	To investigate the effects of vasoactive drugs used in donor care on post-transplant kidney graft function	Noradrenaline decreased the rejection rate and graft loss, presumably by improving hemodynamic stability and organ perfusion
Grigoras et al.^(15)^	Romania	0.806	To investigate the evolution of the potential donor’s hemodynamic status from the BD declaration to organ removal (time in BD)	The interval of hours between BD and harvest appears to have little influence on organ functionality when using early, aggressive, and registered donor management. Functional improvement can be achieved by duration of BD
Isnardi et al.^(16)^	Italy	0.806	To present the use of ECMO as a bridge to organ procurement in a potential donor with hemodynamic instability	ECMO can be used to prevent cardiac arrest, preserve organs, and thus increase the number of potential donors
Jeong et al.^(17)^	South Korea	0.806	To determine the risk factors for organ recovery failure and complications during donor management	BD-related complications were acute kidney injury, cardiopulmonary resuscitation, bacteremia, thrombocytopenia, and *diabetes insipidus* . Success rates of removal and transplantation remained low
Cavalcante et al.^(18)^	Brazil	0.1047	To analyze the opinion of nurses about nursing care for BD patients and potential organ donors	The nurses try to contemplate the technical and bioethical dimensions of care for the potential organ donor and family, although they recognize the complexity of the process and the need for better qualification and emotional maturity
Nicolas-Robin et al.^(19)^	France	5.163	To investigate the benefit of a supplementary dose of hydrocortisone in BD patients in decreasing hemodynamic instability, and the need for noradrenaline	The supplemental dose of hydrocortisone potentiated systemic hemodynamic stability, regardless of the pathophysiological state of the corticosteroids, with or without primary disease or secondary adrenal insufficiency in the patients. Thus, hydrocortisone infusion significantly reduced the dose of noradrenaline required for hemodynamic stability
Mojtabaee et al.^(20)^	Iran	0.806	To describe the pediatric donors, their characteristics, complications, and the organ procurement process; to compare two age groups: under 5 years and those between 5 and 12 years	Organs acquired from pediatric donors can be a valuable and life-saving solution for both children and adults. The different complications in donors under 5 years require attention in the protocols
Pinsard et al.^(21)^	France	6.425	To study the impact of steroid administration on the recovery of function and the number of organs obtained compared to the number of potential BD donors	Early replacement administration of glucocorticoids in an organ BD donor with circulatory failure allows the dose and duration of vasopressor administration to be significantly reduced
Kawati et al.^(22)^	Sweden	1.971	To describe a case of traumatic femur fracture with fat embolism syndrome, which resulted in acute tonsillar herniation and subsequently, BD	Deep sedation and permissive hypercapnia are established recommendations for maintenance of acute respiratory distress syndrome. Furthermore, prone position is an effective method to improve saturation in patients requiring high fractions of inspired oxygen. Intracranial pressure monitoring is a routine procedure to assess cerebral edema
Venkateswaran et al.^(23)^	United Kingdom	3.960	To assess the prevalence of elevated inflammatory markers in potential heart and lung donors; to evaluate the impact of T3 and steroids administered during donor maintenance; to correlate the presence of biomarker levels with donor heart and lung function; and explore the value of these biomarkers in predicting transplant outcome	Therapy with T3 or methylprednisolone do not affect the high prevalence of a pro-inflammatory environment in the organ donor. Elevated procalcitonin and tumor necrosis factor levels are associated with donor cardiac dysfunction. The evaluation of biomarkers can become a warning for intensive maintenance, to restore organs, and in donor maintenance and evaluation, to avoid organ rejection
Tamosuitis et al.^(24)^	Lithuania and the Netherlands	2.170	To evaluate the conjunctival and sublingual microcirculation in BD patients, and compare with healthy volunteers in two opposite conditions: complete arrest *versus* normal arterial blood supply to the brain	Compared to healthy controls, BD patients had significantly reduced conjunctival blood flow and microvascular density. However, the presence of conjunctival flow in the absence of cerebral flow makes it impossible to use the conjunctival microcirculation as a substitute for cerebral flow
Vorlat et al.^(25)^	Belgium	7.955	To evaluate the potential value of BNP measured in a BD donor to predict early cardiac performance in the recipient	Elevated BNP levels in cardiac allograft donors were inversely related to function in transplant recipients. Measuring BNP may be a useful tool in the evaluation of heart donors
Fugate et al.^(26)^	United States	8.055	To characterize hemodynamic patterns after BD	Early knowledge of blood pressure changes is important for clinicians performing BD examinations. Delayed BD declaration in a hemodynamically unstable patient may jeopardize harvest, and therefore, support for maintaining adequate arterial pressures is mandatory
Belzberg et al.^(27)^	United States	0.46	To describe the temporal hemodynamic and oxygen transport patterns of trauma patients, such as those presenting BD	The hyperdynamic state, with exaggerated peripheral perfusion and oxygenation, associated with loss of central vasoconstriction in BD patients, resulted from stress response mechanisms, as opposed to metabolic vasodilation, producing high cardiac output and tissue perfusion. The use of noninvasive hemodynamic monitoring for the measurement of cardiac output and tissue perfusion provides early recognition of flow and perfusion deficits
Nakawaga et al.^(28)^	United States	1.818	To describe the physiology of the Cushing reflex after severe head trauma followed by progressive hypotension refractory to phenylephrine, responsive to vasopressin while awaiting organ procurement	The early use of vasopressin, in addition to an alpha-adrenergic agonist, may be beneficial after BD by providing hemodynamic support, as well as by optimizing the quality of donor organs before transplantation
Giani et al.^(29)^	Italy	15.008	To evaluate the feasibility and efficacy of an apnea testing technique that combines the application of PEEP with subsequent lung recruitment in a large cohort of BD patients	The results of this study, in a large part of consecutive patients, including patients with venoarterial ECMO, demonstrated the strategy of apnea testing associated with the application of PEEP is feasible and practical
Robert et al.^(30)^	France	2.872	To analyze the importance of donor factors, especially the potential role of hemodynamic management relative to delayed function of the transplanted kidney; to analyze urine from organ donors by proton magnetic resonance spectroscopy; and to identify urine markers potentially correlated with delayed renal function in transplanted patients	Donor age, hemodynamic status, and hydroxyethyl starch infusion are risk factors for delayed function of the transplanted kidney

BD: brain death; ECMO: extracorporeal membrane oxygenation; T3: triiodothyronine; BNP: B-type natriuretic peptide; PEEP: positive end expiratory pressure.

The use of drugs as a strategy for hemodynamic maintenance was described in 71.4% (n=15) of articles selected for the integrative review. The main drugs presented are described in table 2 .

**Table 2:** List of drugs and dosage for hemodynamic maintenance in brain death

Author	Dopamine	Epinephrine	Noradrenaline	Dobutamine	Phenylephrine	Hydrocortisone	Methylprednisolone	Vasopressin
Plurad et al.^(10)^			NA*	NA*			NA*	NA*
Dhar et al.^(11)^	5μg/kg/minute		10μg/minute		50μg/minute	300-100mg every 8 hours	15mg/kg	0.5-2U/hour
WestphaI et al.^(12)^	5-10mg/minute	2-10mg/minute	1-2g/kg/minute	10µg/kg/minute				1U initial bolus followed by continuous infusion of 0.5-2.4U/hour
Birtan et al.^(14)^	NA*	NA*	NA*	NA*				NA*
Isnardi et al.^(16)^		2μg/kg/minute	2μg/kg/minute	10µg/kg/minute				
Jeong et al.^(17)^	NA*	NA*		NA*				NA*
Nicolas-Robin et al.^(19)^			0.4μg/kg/minute			50mg		
Pinsard et al.^(21)^						50mg		
Venkateswaran et al.^(23)^							1,000mg	
Tamosuitis et al.^(24)^	2.0-9.0μg/kg/minute		0.04-0.1μg/kg/minute					
Belzberg et al.^(27)^	5g/kg/minute							
Nakawaga et al.^(28)^	3μg/kg/minute				200μg/minute			0.04-0.07U/minute
Giani et al.^(29)^	4.1-12.5mcg/kg/minute	0.10-0.22mcg/kg/minute	0.08-0.19mcg/kg/minute	4.4-6.3mcg/kg/minute				
Robert et al.^(30)^			0.10μg/kg/minute					2mL/kg/hour

* NA: does not present dosage.

A table 3 shows the care for hemodynamic maintenance identified in the studies.

**Table 3:** Hemodynamic maintenance care in brain death

Care	Yes n (%)	No n (%)
Absence of sedation	18 (85.7)	3 (14.3)
Prevention of hypothermia	7 (33.3)	14 (66.7)
Correction of fluid and electrolyte disturbances	17 (81.0)	4 (19.0)
Start or maintenance of enteral diet	2 (9.5)	19 (90.5)
Eye protection with moist gauze	0	21 (100.0)
Noninvasive cardiac monitoring	13 (61.9)	8 (38.1)
Invasive arterial catheter monitoring	10 (47.6)	11 (52.4)
Glycemic control	8 (38.1)	13 (61.9)
Transesophageal echocardiography	8 (38.1)	13 (61.9)
Diuresis control	6 (28.6)	15 (71.4)
Control of ventilation parameters	12 (57.1)	9 (42.9)
Control of central venous pressure	9 (42.9)	12 (57.1)
Control of level of consciousness	5 (23.8)	16 (76.2)
Protective mechanical ventilation	8 (38.1)	13 (61.9)

## DISCUSSION

The identification of 21 studies on hemodynamic maintenance in BD underlines the relevance of this research. This is because prior Brazilian investigations with nurses regarding the care of BD patients demonstrated the limited understanding of the theme, focusing on the technical and bioethical dimensions of care, despite identifying its complexity and need for professional improvement and emotional maturity.^( 8 , 9 , 18 )^

Increasing the dissemination and access of professionals to information about the identification and clinical management of BD patients may contribute to increased number of donors and, in turn, organ donations in health services. Early diagnosis of BD and hemodynamic maintenance contribute to rise in the number of deceased donors.^( 12 )^ Thus, the need for continuing education of professionals is reinforced, since hemodynamic instability and dysfunction are the main exclusion factors for potential organ donors.^( 13 , 26 )^

When reporting the management of potential BD organ donors and the main interventions to maintain hemodynamic stability of these patients, the investigations highlighted the need for well-defined protocols and hemodynamic standards, to guide the team and support the care of BD patients. As an example, one study stated the interval between the BD declaration and organ harvesting had little influence on organ quality, by applying an early aggressive protocol in the management of these potential donors, i.e., the fast and effective implementation of actions for the maintenance of organic functions.^( 15 )^

Clinical protocols and guidelines contribute to define and clarify the appropriate care for potential organ donors, to know the real situation of the patient (living or dead); pass on safe information; avoid useless therapy (treating a corpse); reduce costs; improve intensive care beds, and offer the family the option of helping other people through organ and tissue donation.^( 1 )^ Given these facts, the need to implement measures for the maintenance of the potential organ donor is reaffirmed. These measures are employed during the BD verification process, and while waiting for the interview with the family to confirm donation.

Regarding the use of drugs as a form of hemodynamic maintenance, the listed articles mentioned absence of sedation, which corroborates the Manual for Reporting, Diagnosis of Brain Death, and Maintenance of the Potential Organ and Tissue Donor^( 1 )^, which stresses the importance of discontinuing sedative drugs in potential donors, since they act on the central nervous system, depressing and possibly changing the neurological examination assessment in diagnosis of BD.^( 1 )^ It is noteworthy that the first neurological examination should be performed after a minimum interval of four to five half-lives after drug discontinuation.^( 6 )^ Given the natural waiting period for death determination, family approach, and organ harvesting, the use of vasoactive drugs is indicated to hemodynamically stabilize the patient and increase organ transplantation rates.^( 1 )^

Several physiological and systemic disorders develop after BD. For example, central *diabetes insipidus* may occur in 80% of cases with hypothalamic-pituitary dysfunction and deficiency in the production of the antidiuretic hormone. The absence of this hormone may result in progressive clinical manifestations, such as hypoosmolar polyuria, secondary hypovolemia, hypernatremia, and serum hyperosmolarity.^( 12 )^In view of this, some studies^( 10 , 28 )^showed the use of vasopressin in increasing the rate of organ recovery, besides improving the quality of the organs. This is justified by its excellent vasoconstrictor and antidiuretic effect acting on vascular smooth muscle V1 receptors, V2 receptors, present in the renal collecting system and that increase fluid reabsorption due to increased serum osmolarity, and V3 receptors, which regulate corticotrophin levels.^( 10 )^In addition, early vasopressin administration optimizes hemodynamic support to BD patients.^( 28 )^

The importance of managing endocrine-metabolic disorders should be emphasized, since they are directly related to hemodynamic stability of the potential donor, and their complications can lead to severe disorders and ineffective organ donation.^( 12 )^ Therefore, hypernatremia, characterized by high sodium concentrations (>145mEq/L) in the body, is associated as a risk factor for organ donation, since it presents inefficient results after transplantation, despite conflicting evidence. Therefore, a study proposing the *in vitro* analysis of pancreatic islets from donors who had hypernatremia, concluded there is functional impairment of the organ when transplanted to the recipient.^( 31 )^

Another study showed hypernatremia prior to liver transplantation is related to a significant post-transplantation mortality rate.^( 32 )^On the other hand, a study conducted with pediatric donors shows that there are no negative results when related to hypernatremia immediately prior to organ harvesting.^( 33 )^ Yet, another investigation associated increased catecholamine infusion with a favorable 4-year survival rate in patients who received kidney transplants;^( 14 )^ among the catecholamines found in this investigation, noradrenaline and dopamine stood out.

According to the Manual for Notification, Diagnosis of Brain Death and Maintenance of Potential Organ and Tissue Donor,^( 1 )^ if the patient presents hypotension, norepinephrine is the first choice of vasoactive drug to reach adequate blood pressure levels, where the ideal is to maintain mean arterial pressure >65mmHg or systolic >90mmHg.^( 34 )^ The noradrenaline dose recommended by the manual is 0.05 to 2mcg/kg/minute^1^ ; in the articles searched, it ranged from 0.4μg/kg/minute to 0.10μg/kg/minute, demonstrating the need for a higher dose than that indicated to maintain stability in the patients studied. Revision of the manual may be required.

The articles point to the beneficial effects of norepinephrine and attest to the significant effect on hemodynamic stability of potential BD donors,^( 1 )^ and on reducing organ rejection rates.^( 14 )^ In addition, the infusion of catecholamines, such as norepinephrine, can be adjusted, with the purpose of maintaining mean arterial pressure above 65mmHg, and making organs viable for donation.^( 30 , 35 )^ However, in cases of mixed neurogenic and hypovolemic shock, dopamine infusion up to 10µg/kg/minute is indicated, avoiding dobutamine with increased oxygen consumption and noradrenaline, because it causes vasoconstriction,^( 36 )^ thus requiring a detailed evaluation by the healthcare professional to identify the basic causes and determine the assertive management.

The administration of corticosteroids, such as hydrocortisone, also enhances hemodynamic stability and can reduce the accumulated volume in cases of BD with circulatory failure, since it acts as an adrenergic blocker in patients with some adrenal deficiency.^( 19 , 21 )^ Methylprednisolone is recommended for its anti-inflammatory action in liver grafts, associated with reduction in post-transplant liver inflammation.^( 12 )^ A comparative study between high dose (methylprednisolone) and low dose (hydrocortisone) corticosteroids identified the use of low doses corticosteroids did not result in worsening of the lung or heart condition, with transplanted organs compared to the traditional use of methylprednisolone.^( 11 )^

The use of corticosteroids is based on common findings, and their combination with vasoactive drugs has resulted in significant improvement in organ procurement.^( 11 , 34 )^A multicenter prospective controlled study showed that the administration of low-dose steroids provides weaning from vasopressors, and also reduces the amount of vasopressors needed to control circulatory failure by more than 20%, allowing a significant reduction in the need for inotropic support,^( 21 )^ which reinforces the use of the drug as an ally to hemodynamic management in BD.

Regarding the care and control essential to maintain the potential donor, a protocol of guidelines for care considers some actions as essential, such as body temperature limits, blood pressure limits, and control of biochemical markers. According to the protocol, BD paralyzes hypothalamic thermoregulatory functions, leading to progressive hypothermia, which demonstrates the importance of body temperature regulation, since it provides thermal homeostasis. It is recommended to maintain body temperature above 35^o^C (ideally between 36C^o^ and 37.5C^o^) with use of thermal blankets, mechanical ventilation, and warmed intravenous fluid infusion.^( 12 )^

The main challenge in the management of potential donors is hemodynamic instability, which points to the need for comprehensive knowledge about the organic processes involving BD, and the procedures indicated to mitigate such changes. This is due to the fact that many patients have prolonged hypoperfusion, i.e., after BD there is adrenergic hyperactivity, leading to tachycardia, increased systemic vascular resistance, increased myocardial oxygen consumption, and systolic reflex of hypertension.^( 12 )^

The first event, prolonged hypoperfusion, classified by the author as “sympathetic storm,” lasts from 20 to 30 minutes, and is followed by hypotension. Subsequently, the second phase, characterized as “adrenergic storm,” produces hypertension, causing hypoperfusion due to transient vasoconstriction.^( 12 )^ Noninvasive hemodynamic monitoring to measure cardiac output and tissue perfusion allows the identification of these phases,^( 27 )^ and thus, early intervention, aiming at organ preservation.

There are significant differences between invasive and noninvasive monitoring, especially when exacerbated vasoconstriction occurs. In this case, invasive measures for blood pressure maintenance are recommended, since they are safer and easier for collecting samples, through an arterial blood gas test, as well as blood pressure determination.^( 12 )^Moreover, due to hemodynamic instability and possible complications, ECMO may be used as a strategy to prevent cardiac arrest and preserve organs.^( 16 )^

Some procedures rarely mentioned, such as the start or maintenance of an enteral diet and eye protection with moist gauze, may be justified by the fact that most articles addressed drug issues and other specific care for hemodynamic stability. However, the manual recommends maintaining the diet to supply 15% to 30% of daily needs, but discontinuing its infusion if there are increased doses of vasoactive drugs and signs of tissue hypoperfusion.^( 1 )^ This is a crucial care for maintenance of tissue nutrition.

Although the search was limited to articles with free access in the databases and through the listed descriptors, it was observed that ensuring hemodynamic maintenance in BD is essential for the viability of the transplant. To this end, it is necessary that the team of healthcare professionals who participate in this process be aware of the clinical conditions, and all aspects that involve care and management in BD. Therefore, complying with and always updating the protocols based on findings of the literature are of utmost importance for better outcomes on this topic.

## CONCLUSION

The main strategies for hemodynamic maintenance of the potential organ donor are based on the control of cardiovascular and respiratory parameters, as well as drug interventions.
