# Supplementary material for: Strategies for hemodynamic maintenance of potential brain-dead donor: integrative review
Source: Einstein (Sao Paulo). 2021 Jun 17;19:eRW5630. doi: 10.31744/einstein_journal/2021RW5630 (PMC8225261; doi:10.31744/einstein_journal/2021RW5630)
Supplement: Translation

# Translation: Estratégias para manutenção hemodinâmica do potencial doador em morte encefálica: revisão integrativa

**Authors:** Beatriz Sousa da Fonseca, Verusca Soares de Souza, Taynara Oliveira Farias Batista, Guilherme Malaquias Silva, Dandara Novakowski Spigolon, Neide Derenzo, Aline Barbieri

**Affiliations:** 1 Brasil Universidade Estadual do Paraná, Paranavaí, PR, Brasil.; 2 Brasil Universidade Federal de Mato Grosso do Sul, Coxim, MS, Brasil.

**Keywords:** Morte encefálica, Hemodinâmica, Cuidados de enfermagem, Transplante de órgãos

## Abstract

**Objetivo:**

Conhecer a produção científica sobre estratégias adotadas para a manutenção hemodinâmica de pacientes com morte encefálica.

**Métodos:**

Revisão integrativa com artigos publicados entre 2007 e 2019, nas bases *Scientific Electronic Library Online* (SciELO), Literatura Latino-Americana e do Caribe em Ciências da Saúde (LILACS), PubMed^®^e *ScienceDirect* . Utilizaram-se os termos “Hemodinâmica *AND* Morte Encefálica” e *“Hemodynamics AND Brain Death* .” Os critérios de exclusão foram pesquisas não realizadas com seres humanos e literatura cinzenta.

**Resultados:**

Listaram-se 21 artigos. Como estratégias, destacaram-se uso de medicamentos – noradrenalina (n=8), vasopressina (n=7), dobutamina (n=6), hidrocortisona (n=4) e metilprednisolona (n=4); monitorização cardíaca invasiva (n=10) e não invasiva (n=13); controle dos parâmetros ventilatórios (n=12); e correção de distúrbios hidroeletrolíticos (n=17).

**Conclusão:**

As principais estratégias encontradas nesta revisão integrativa foram regulação da pressão arterial e temperatura, uso de catecolaminas e corticosteroides, além da necessidade de um diagnóstico precoce de morte encefálica. Entretanto, é notória a escassez de protocolos mais esclarecidos sobre a temática, dificultando o manejo com o potencial doador.

## INTRODUÇÃO

O transplante de órgãos é a alternativa terapêutica mais eficaz em muitos pacientes com doenças terminais, e a fila de espera para transplantes tem aumentado ano após ano. Conflitos éticos e percepção familiar têm sido estudados, observando aumento gradativo de pesquisas relacionadas a essa temática desde 2010, o que demonstra a necessidade de uma análise reflexiva para que o processo de doação de órgãos seja elucidado.^( 1 )^

Reforça-se, então, a educação em saúde como aspecto relevante para a propagação de informações sobre a doação de órgãos e a morte encefálica (ME), o que evidencia a importância da participação da equipe multiprofissional de saúde.^( 2 )^No entanto, a carência de evidências mais abrangentes sobre o tema denota a demanda de orientações mais claras para que se proporcione uma uniformidade nas condutas com os indivíduos que necessitam desse cuidado. Para tal, é importante que se considerem os aspectos clínicos e bioéticos.^( 3 )^

O Brasil é o segundo país do mundo em número de transplantes, atrás dos Estados Unidos.^( 4 )^Houve aumento de apenas 2,4% de doadores efetivos em 2018, passando de 16,6 por milhão de população (pmp), em 2017, para 17,0 pmp, em 2018. Esse aumento foi justificado pela elevação da taxa de notificação de potenciais doadores em 2,2% da taxa de concretização da doação. A taxa de recusa familiar se manteve em 43%, sendo inferior no Paraná (27%) e superior no Mato Grosso (80%). Em 2019, o número de doadores efetivos foi de 18,1 pmp, com 40% de recusa familiar.^( 4 )^

Apesar disso, é notória a carência brasileira na pesquisa e no aprofundamento da temática. A produção e a publicação sistematizada e detalhada contribuiriam para a ocorrência de transplantes e auxiliariam no processo de diagnóstico de ME, na manutenção de potenciais doadores e com subsídios em educação para profissionais e familiares.^( 5 )^

A ME é caracterizada pela perda completa e irreversível das funções encefálicas, definidas pela cessação das atividades corticais e de tronco encefálico.^( 6 , 7 )^O Conselho Federal de Medicina (CFM), em seu protocolo, instituído pela Resolução 2.173 de 2017, determina que, para passar pelos procedimentos diagnósticos de determinação de ME, o paciente deve apresentar todos os seguintes pré-requisitos: presença de lesão encefálica de causa conhecida e irreversível; ausência de fatores tratáveis que confundiriam o diagnóstico; tratamento e observação no hospital pelo período mínimo de 6 horas; temperatura corporal superior a 35^o^C e saturação arterial de acordo com critérios estabelecidos pela Resolução.^( 6 )^

A ME e o transplante de órgãos e tecidos estão atrelados, visto que, com a ME, o paciente pode se tornar um doador múltiplo de órgãos em potencial, o que depende de seu estado geral.^( 8 )^Dessa forma, garantir a manutenção funcional dos sistemas orgânicos no período transcorrido entre o diagnóstico e a entrevista familiar se torna principal objetivo da equipe de saúde, no intuito de impedir o comprometimento de órgãos e impossibilitar a doação.

As ações relacionadas à comunicação do óbito à Central Estadual de Transplante e ao apoio na manutenção do potencial doador pelos profissionais da terapia intensiva são responsabilidades da Comissão Intra-Hospitalar de Doação de Órgãos e Tecidos para Transplantes (CIHDOTT) e da Organização de Procura de Órgãos (OPO).^( 1 )^Dessa forma, ressalta-se a importância da interdisciplinaridade e do trabalho em equipe, para que o processo seja efetivo no manejo das repercussões fisiopatológicas próprias da ME, na monitorização hemodinâmica e na prestação de cuidados individualizados.

Determinada a importância do controle hemodinâmico na manutenção do potencial doador de órgãos na ocorrência da ME, questiona-se: Quais as estratégias frequentemente adotadas para a manutenção hemodinâmica em pacientes em ME?

## OBJETIVO

Conhecer a produção científica sobre as estratégias adotadas para a manutenção hemodinâmica de potenciais doadores em morte encefálica.

## MÉTODOS

Trata-se de uma de revisão integrativa, desenvolvida de acordo com os seguintes passos: identificação do tema e elaboração da questão de pesquisa; estabelecimento dos critérios de inclusão e exclusão; busca e categorização dos estudos; avaliação dos artigos incluídos; interpretação dos resultados; síntese do conhecimento e apresentação da revisão.^( 9 )^

A busca foi realizada no período de novembro de 2018 a fevereiro de 2019, guiada pela questão norteadora: Quais as estratégias frequentemente adotadas para a manutenção hemodinâmica em pacientes em ME? As bases de dados eletrônicas escolhidas para a busca dos artigos foram Literatura Latino-Americana e do Caribe em Ciências da Saúde (LILACS), PubMed®, *Scientific Electronic Library Online* (SciELO), e *ScienceDirect* . Como descritores, utilizaram-se: “Hemodinâmica *AND* Morte Encefálica”, dos Descritores em Ciências da Saúde (DeCS), e “ *Hemodynamics AND Brain Death* ”, do *Medical Subject Headings* (MeSH).

Foram incluídos artigos com disponibilidade pela universidade ou gratuitos e na íntegra; nos idiomas português, inglês ou espanhol e publicados entre os anos de 2007 e 2019. Excluíram-se os estudos que não foram realizados com seres humanos, não fossem artigo científico e os repetidos nas bases de dados. O fluxo de seleção está descrito na figura 1 .

**Figura 1:** Fluxograma da busca na literatura

LILACS: Literatura Latino-Americana e do Caribe em Ciências da Saúde; SciELO: *Scientific Electronic Library Online* .

Para análise dos artigos, utilizou-se instrumento próprio, que extraiu informações relacionadas a autores, periódico, ano de publicação, Fator de Impacto, objetivo e conclusão. Essas informações das publicações foram apresentadas por meio de quadro-síntese.

De forma complementar, elaborou-se um *checklist* , organizado em resposta dicotômica (sim e não), pautado na ficha de manutenção hemodinâmica disponibilizada às CIHDOTT do Paraná, que inclui alguns cuidados com o paciente em ME: ausência de sedação, prevenção de hipotermia, correção de distúrbios hidroeletrolíticos, início/manutenção de dieta enteral, proteção ocular, monitorização cardíaca não invasiva ou invasiva, controle glicêmico, ecocardiograma transoesofágico, controle de parâmetros ventilatórios, controle de pressão venosa central, controle de nível de consciência e ventilação mecânica protetora. Ademais, todos os itens apontados pelos artigos como ação de manutenção hemodinâmica do potencial doador foram incluídos na coleta, mesmo que não contemplados pelo instrumento elaborado inicialmente, como as medicações e sua respectiva indicação de dose.

Os artigos foram enumerados por ordem de seleção e, para os cuidados indicados, procedeu-se à análise de frequência e percentagem.

## RESULTADOS

A síntese dos 21 artigos que compõem este estudo, acrescida de seu país de origem e do Fator de Impacto do periódico, é apresentada na tabela 1 . Dos 21 artigos listados, 95,2% (n=20) foram publicados em periódicos de origem internacional e apenas um em periódico brasileiro. Entre os artigos estrangeiros selecionados, 23,8% (n=5) pertenciam aos Estados Unidos; 14,2% (n=3) à França e 9,5% (n=2) à Itália. Uruguai, Romênia, Irã, Reino Unido, Turquia, Coreia do Sul, Bélgica, Suécia, Lituânia e Holanda totalizaram 42,8%, tendo cada país um artigo.

**Tabela 1:** Síntese dos artigos acerca da manutenção hemodinâmica na morte encefálica

Autor	País	Fator de impacto	Objetivo	Conclusão
Plurad et al.^(10)^	Estados Unidos	2,403	Determinar o efeito da arginina vasopressina nas taxas de recuperação de órgãos	O uso de arginina vasopressina com terapia de reposição hormonal é associado a aumento na taxa de recuperação de órgãos. Essa estratégia deve ser adotada universalmente na manutenção de doadores
Dhar et al.^(11)^	Estados Unidos	2,872	Comparar um protocolo de esteroide de dose mais baixa (adequado para estabilização da insuficiência adrenal e sepse) ao regime tradicional de altas doses na manutenção de doadores de órgãos com ME	Um protocolo de corticosteroide de dose mais baixa não resultou em piora da função pulmonar ou cardíaca do doador, em comparação com o regime tradicional de altas doses. As necessidades de insulina e controle glicêmico foram melhoradas. A metilprednisolona em altas doses pode não ser necessária para apoiar doadores com ME
WestphaI et al.^(12)^	Brasil	0,806	Estabelecer diretrizes para o cuidado e a manutenção de adultos doadores de órgãos, orientando e padronizando os cuidados prestados a potenciais doadores em ME	Foi desenvolvido protocolo com ações consideradas essenciais para manutenção dos doadores de órgãos: limites da temperatura corporal, exames laboratoriais indicados, limites de pressão arterial e uso de catecolaminas
Godino et al.^(13)^	Uruguai	0,806	Identificar e quantificar as causas de exclusão de potenciais doadores de coração, e definir os fatores de risco para disfunção ventricular entre a população de ME	A disfunção hemodinâmica em jovens com traumatismo craniano representou a principal causa de perda de doadores de coração. O uso de vasopressores foi observado em todos os pacientes investigados
Birtan et al.^(14)^	Turquia	0,806	Investigar os efeitos de drogas vasoativas utilizadas no cuidado de doadores nas funções do enxerto renal pós-transplante	A noradrenalina diminuiu a taxa de rejeição e a perda do enxerto, presumivelmente melhorando a estabilidade hemodinâmica e a perfusão de órgãos
Grigoras et al.^(15)^	Romênia	0,806	Investigar a evolução do estado hemodinâmico do potencial doador desde a declaração de ME até a remoção dos órgãos (tempo em ME)	O manejo intensivo de pacientes com ME é fundamental para a qualidade dos órgãos colhidos e evolução do enxerto pós-transplante. O intervalo entre a declaração de ME e a colheita dos órgãos parecia ter pouca influência na qualidade dos órgãos colhidos, usando um protocolo de manejo agressivo do doador durante a duração do ME.
Isnardi et al.^(16)^	Itália	0,806	Apresentar o uso da ECMO como ponte para a obtenção de órgãos em um doador em potencial com instabilidade hemodinâmica	A ECMO pode ser usada para prevenir a parada cardíaca, preservar os órgãos e, assim, aumentar o número de potenciais doadores
Jeong et al.^(17)^	Coreia do Sul	0,806	Determinar os fatores de risco para falha na recuperação de órgãos e complicações durante o manejo de doadores	As complicações relacionadas à ME foram lesão renal aguda, ressuscitação cardiopulmonar, bacteremia, trombocitopenia e diabetes insípido. As taxas de sucesso de remoção e transplante permaneceram baixas
Cavalcante et al.^(18)^	Brasil	0,1047	Analisar a opinião dos enfermeiros sobre os cuidados de enfermagem voltados a paciente em ME e potencial doador de órgãos	Os enfermeiros buscam contemplar as dimensões técnica e bioética do cuidado voltado ao paciente potencial doador de órgãos e à sua família, embora reconheçam a complexidade do processo e a necessidade de melhor qualificação e maturidade emocional
Nicolas-Robin et al.^(19)^	França	5,163	Investigar o benefício da dose suplementar de hidrocortisona em pacientes com ME, na diminuição da instabilidade hemodinâmica e da necessidade de noradrenalina	A dose suplementar de hidrocortisona potencializou a estabilidade hemodinâmica sistêmica, independente do estado fisiopatológico dos corticoides, com ou sem doença primária ou insuficiência adrenal secundária nos pacientes. Dessa forma, a infusão de hidrocortisona reduz significativamente a dose da noradrenalina necessária para a estabilidade hemodinâmica
Mojtabaee et al^.(20)^	Irã	0,806	Descrever os doadores pediátricos, suas características, complicações e processo de obtenção dos órgãos; comparar dois grupos etários: menores de 5 anos e aqueles entre 5 e 12 anos de idade	Órgãos adquiridos de doadores pediátricos podem ser uma solução valiosa e salvar vidas de crianças e adultos. As diferentes complicações em doadores menores de 5 anos requerem atenção nos protocolos
Pinsard et al.^(21)^	França	6,425	Estudar o impacto da administração de esteroides na recuperação da função e no número de órgãos obtidos em comparação com o número de potenciais doadores com ME	A administração substitutiva precoce de glicocorticoides em um doador de órgãos em ME com insuficiência circulatória, permite reduzir significativamente a dose e a duração da administração de vasopressores
Kawati et al.^(22)^	Suécia	1,971	Descrever um caso de fratura traumática de fêmur com síndrome de embolismo gorduroso, que gerou herniação tonsilar aguda e, posteriormente, ME	Sedação profunda e hipercapnia permissiva são recomendações estabelecidas para a manutenção de síndrome do desconforto respiratório agudo. Ademais, posição prona é um método efetivo para melhorar a saturação em pacientes com necessidade de altas frações inspiradas de oxigênio. Verificar pressão intracraniana é procedimento de rotina para monitorar edema cerebral
Venkateswaran et al.^(23)^	Reino Unido	3,960	Avaliar a prevalência de marcadores inflamatórios elevados em potenciais doadores de coração e pulmão; avaliar o impacto da T3 e dos esteroides administrados durante a manutenção de doadores; correlacionar a presença de níveis de biomarcadores com a função cardíaca e pulmonar de doadores; e explorar o valor desses biomarcadores na previsão do resultado do transplante	Existe alta prevalência de um ambiente pró-inflamatório no doador de órgãos que não é afetado com terapia T3 ou metilprednisolona. Níveis elevados de procalcitonina e fator de necrose tumoral estão associados à disfunção cardíaca do doador. A avaliação dos biomarcadores pode tornar-se um alerta para uma manutenção intensiva, para restaurar órgãos e na manutenção e avaliação do doador, evitando-se rejeição de órgãos
Tamosuitis et al.^(24)^	Lituânia e Holanda	2,170	Avaliar a microcirculação conjuntival e sublingual em pacientes em ME, e comparar com voluntários saudáveis em duas condições opostas: parada completa *versus* fornecimento normal de sangue arterial ao cérebro	Em comparação com controles saudáveis, pacientes em ME tiveram redução significativa no fluxo sanguíneo e densidade microvascular conjuntival. Entretanto, a presença de fluxo conjuntival na ausência de fluxo cerebral torna impossível usar a microcirculação conjuntival como substituta do fluxo cerebral
Vorlat et al.^(25)^	Bélgica	7,955	Avaliar o valor potencial do BNP medido em doador com ME para prever o desempenho cardíaco precoce no receptor	Níveis elevados de BNP em doadores de aloenxerto cardíaco foram inversamente relacionados com a função nos receptores de transplantes. Mensurar BNP pode se tornar ferramenta útil na avaliação dos doadores de coração
Fugate et al.^(26)^	Estados Unidos	8,055	Caracterizar padrões hemodinâmicos após a ME	O conhecimento antecipado das mudanças na pressão arterial é importante para os médicos que realizam os exames de ME. Atraso na declaração de ME em um paciente hemodinamicamente instável pode colocar em risco a aquisição, e, por isso, o apoio para manter pressões arteriais adequadas é obrigatório
Belzberg et al.^(27)^	Estados Unidos	0,46	Descrever os padrões hemodinâmicos temporais e de transporte de oxigênio de pacientes com traumatismos, como os padrões daqueles em ME	O estado hiperdinâmico, com a perfusão e a oxigenação periférica exagerada, associada à perda da vasoconstrição central em paciente em ME, resultou dos mecanismos de resposta ao estresse, em oposição à vasodilação metabólica, produzindo alto débito cardíaco e perfusão tecidual. O uso de monitorização hemodinâmica não invasiva para a mensuração do débito cardíaco e perfusão tecidual fornece reconhecimento precoce de deficiências de fluxo e perfusão
Nakawaga et al.^(28)^	Estados Unidos	1,818	Descrever a fisiologia do reflexo de Cushing após traumatismo craniencefálico grave seguido de hipotensão progressiva refratária à fenilefrina, responsiva à vasopressina durante a espera pela obtenção dos órgãos	O uso precoce de vasopressina, além de um agonista alfa-adrenérgico, pode ser benéfico após a ME, por fornecer suporte hemodinâmico, como também por otimizar a qualidade dos órgãos do doador antes do transplante
Giani et al.^(29)^	Itália	15,008	Avaliar a viabilidade e a eficácia de uma técnica de teste de apneia que combina a aplicação de PEEP com recrutamento pulmonar subsequente em uma grande coorte de pacientes em ME	Os resultados deste estudo, em grande parte de pacientes consecutivos, incluindo pacientes com ECMO venoarterial, demonstram que a estratégia de teste de apneia associada com a aplicação de PEEP é viável e prática
Robert et al.^(30)^	França	2,872	Analisar a importância dos fatores do doador, especialmente o papel potencial do manejo hemodinâmico, em relação ao atraso da função do rim transplantado; analisar a urina de doadores de órgãos por espectroscopia de prótons por ressonância magnética; identificar marcadores de urina potencialmente correlacionados com o atraso da função renal em pacientes transplantados	Idade do doador, *status* hemodinâmico e infusão de amido de hidroxietil são fatores de risco para atraso na função do rim transplantado

ME: morte encefálica; ECMO: oxigenação por membrana extracorpórea; T3: triioditironina; BNP: peptídeo natriurético do tipo B; PEEP: pressão positiva expiratória final.

O uso de medicamentos como estratégia de manutenção hemodinâmica foi descrito em 71,4% (n=15) dos artigos selecionados para a revisão integrativa. As principais drogas apresentadas estão descritas na tabela 2 .

**Tabela 2:** Relação de medicamentos e dosagem para a manutenção hemodinâmica na morte encefálica

Autor	Dopamina	Epinefrina	Noradrenalina	Dobutamina	Fenilefrina	Hidrocortisona	Metilpredinisolona	Vasopressina
Plurad et al.^(10)^			NA*	NA*			NA*	NA*
Dhar et al.^(11)^	5μg/kg/minuto		10μg/minuto		50μg/minuto	300-100mg a cada 8 horas	15mg/kg	0,5-2U/hora
WestphaI et al.^(12)^	5-10mg/minuto	2-10mg/minuto	1-2g/kg/minuto	10µg/kg/minuto				1U bólus inicial seguido por contínua infusão de 0,5-2,4U/hora
Birtan et al.^(14)^	NA*	NA*	NA*	NA*				NA*
Isnardi et al.^(16)^		2μg/kg/minuto	2μg/kg/minuto	10µg/kg/minuto				
Jeong et al.^(17)^	NA*	NA*		NA*				NA*
Nicolas-Robin et al.^(19)^			0,4μg/kg/minuto			50mg		
Pinsard et al.^(21)^						50mg		
Venkateswaran et al.^(23)^							1.000mg	
Tamosuitis et al.^(24)^	2,0-9,0μg/kg/minuto		0,04-0,1μg/kg/minuto					
Belzberg et al.^(27)^	5g/kg/minuto							
Nakawaga et al.^(28)^	3μg/kg/minuto				200μg/minuto			0,04-0,07U/minuto
Giani et al.^(29)^	4,1-12,5mcg/kg/minuto	0,10-0,22mcg/kg/minuto	0,08-0,19mcg/kg/minuto	4,4-6,3mcg/kg/minuto				
Robert et al.^(30)^			0,10μg/kg/minuto					2mL/kg/hora
* NA: não apresenta dosagem.								

A tabela 3 apresenta os cuidados para a manutenção hemodinâmica identificados nos estudos.

**Tabela 3:** Cuidados de manutenção hemodinâmica na morte encefálica

Cuidados	Sim n (%)	Não n (%)
Ausência de sedação	18 (85,7)	3 (14,3)
Prevenção de hipotermia	7 (33,3)	14 (66,7)
Correção de distúrbios hidroeletrolíticos	17 (81,0)	4 (19,0)
Início ou manutenção de dieta enteral	2 (9,5)	19 (90,5)
Proteção ocular com gaze úmida	0	21 (100,0)
Monitorização cardíaca não invasiva	13 (61,9)	8 (38,1)
Monitorização invasiva por cateter arterial	10 (47,6)	11 (52,4)
Controle glicêmico	8 (38,1)	13 (61,9)
Ecocardiograma transoesofágico	8 (38,1)	13 (61,9)
Controle de diurese	6 (28,6)	15 (71,4)
Controle dos parâmetros ventilatórios	12 (57,1)	9 (42,9)
Controle de pressão venosa central	9 (42,9)	12 (57,1)
Controle de nível de consciência	5 (23,8)	16 (76,2)
Ventilação mecânica protetora	8 (38,1)	13 (61,9)

## DISCUSSÃO

A identificação dos 21 estudos acerca da manutenção hemodinâmica na ME reforça a pertinência desta investigação. Isso porque investigações brasileiras anteriores junto a enfermeiros em relação aos cuidados com pacientes em ME salientaram a compreensão limitada sobre a temática, com foco no atendimento às dimensões técnicas e bioéticas do cuidado, apesar de identificarem sua complexidade e necessidade de aprimoramento profissional e maturidade emocional.^( 8 , 9 , 18 )^

Aumentar a divulgação e o acesso dos profissionais a informações acerca da identificação e do manejo clínico do paciente em ME talvez contribua ao aumento do número de doadores e, por sua vez, de doações de órgãos nos serviços de saúde. O diagnóstico precoce da ME e a manutenção hemodinâmica contribuem para o aumento do número de doadores falecidos.^( 12 )^Desse modo, reforça-se a necessidade da educação permanente dos profissionais, visto que a instabilidade e a disfunção hemodinâmica são os principais fatores de exclusão de potenciais doadores de órgãos.^( 13 , 26 )^

Ao relatar o manejo com potenciais doadores de órgãos em ME e as principais intervenções para manter a estabilidade hemodinâmica desses pacientes, as investigações ressaltaram a necessidade de protocolos e padrões hemodinâmicos bem definidos, com o intuito de direcionar a equipe e subsidiar os cuidados com pacientes em ME. A exemplo disso, um estudo afirmou que o intervalo entre a declaração da ME e a captação de órgãos teve pouca influência na qualidade dos órgãos, pela aplicação de um protocolo agressivo precoce no manejo desses potenciais doadores, ou seja, a implementação de forma rápida e eficaz de ações para a manutenção das funções orgânicas.^( 15 )^

Protocolos e diretrizes clínicas contribuem para definição e esclarecimento dos cuidados adequados com potenciais doadores de órgãos, a fim de conhecer a real situação do paciente (vivo ou morto); passar informações seguras; evitar terapia inútil (tratar cadáver); reduzir custos; aperfeiçoar leitos de terapia intensiva e oferecer à família a opção de ajudar outras pessoas por meio da doação de órgãos e tecidos.^( 1 )^Diante desses fatos, reafirma-se a necessidade de implantação de medidas para a manutenção do potencial doador de órgãos, instituídas durante o processo de certificação da ME e enquanto se aguarda a entrevista familiar para concretizar a doação.

Em relação ao uso de medicamentos como forma de manutenção hemodinâmica, os artigos listados citavam ausência da utilização de sedação, o que corrobora o Manual para Notificação, Diagnóstico de Morte Encefálica e Manutenção do Potencial Doador de Órgãos e Tecido, que destaca a importância da suspensão de drogas sedativas em potenciais doadores, já que agem no sistema nervoso central deprimindo e podendo alterar a avaliação do exame neurológico no diagnóstico de ME.^( 1 )^Ressalta-se que o primeiro exame neurológico deve ser realizado a partir de um intervalo mínimo de quatro a cinco meias-vidas após a suspensão dos fármacos.^( 6 )^Diante da necessidade natural de espera para determinação do óbito, abordagem familiar e captação do órgão, indica-se o uso de drogas vasoativas, a fim de estabilizar o paciente hemodinamicamente e elevar as taxas de órgãos para transplante.^( 1 )^

A partir da ME, diversos distúrbios fisiológicos e sistêmicos se desenvolvem. A exemplo disso, com a disfunção hipotálamo-hipofisária e a deficiência na produção do hormônio antidiurético, pode ocorrer *diabetes insipidus* central, presente em 80% dos casos. A ausência desse hormônio pode resultar em manifestações clínicas progressivas, como poliúria hiposmolar, hipovolemia secundária, hipernatremia e hiperosmolaridade sérica.^( 12 )^Diante disso, alguns estudos^( 10 , 28 )^evidenciam o uso de vasopressina na elevação da taxa de recuperação dos órgãos, além de aperfeiçoar a qualidade dos mesmos. Isso é justificado por seu excelente efeito vasoconstritor e antidiurético, atuando sobre receptores V1 do músculo liso vascular; receptores V2, presentes no sistema coletor renal e que aumentam a reabsorção de fluidos devido à elevação da osmolaridade sérica, e receptores V3, que regulam os níveis de corticotrofina.^( 10 )^Além disso, a vasopressina administrada precocemente otimiza o suporte hemodinâmico ao paciente com ME.^( 28 )^

Vale salientar a importância do manejo com os distúrbios endócrino-metabólicos, já que estão diretamente ligados com a estabilidade hemodinâmica do potencial doador, e sua complicação pode acarretar transtornos severos e ineficácia na doação de órgãos.^( 12 )^Para tanto, a hipernatremia, caracterizada por concentrações elevadas de sódio (>145mEq/L) no organismo, é associada como fator de risco para a doação de órgãos, visto que apresenta resultados ineficientes após os transplantes, apesar de existirem evidências conflitantes. Diante disso, um estudo que propôs a análise in vitro de ilhotas pancreáticas de doadores que apresentaram hipernatremia concluiu que há diminuição funcional do órgão quando transplantado para o receptor.^( 31 )^

Outro estudo evidencia que a hipernatremia anterior ao transplante de fígado está relacionada com taxa significativa de mortalidade pós-transplante.^( 32 )^Em contrapartida, estudo realizado com doadores pediátricos revela que não houve resultados negativos quando relacionados à hipernatremia imediata anterior a captação do órgão.^( 33 )^Ainda, outra investigação associou aumento na infusão de catecolaminas com o favorecimento na taxa de sobrevida em 4 anos de pacientes que receberam transplantes renais;^( 14 )^entre as catecolaminas encontradas na presente investigação, destacaram-se a noradrenalina e a dopamina.

De acordo com Manual para Notificação, Diagnóstico de Morte Encefálica e Manutenção do Potencial Doador de Órgãos e Tecido^( 1 )^se o paciente apresentar hipotensão, a noradrenalina é a droga vasoativa de primeira escolha, a fim de atingir os níveis adequados da pressão arterial, em que o ideal é manter pressão arterial média >65mmHg ou sistólica >90mmHg.^( 34 )^A dose de noradrenalina recomendada pelo manual é de 0,05 a 2mcg/kg/minuto^1^ ; já nos artigos encontrados, variou entre 0,4μg/kg/minuto a 0,10μg/kg/minuto, o que demonstra necessidade de dosagem maior do que a indicada para a manutenção da estabilidade dos pacientes investigados, podendo gerar necessidade de revisão do manual.

Os artigos apontam para efeitos benéficos da noradrenalina e atestam o efeito significativo da estabilidade hemodinâmica de potenciais doadores em ME,^( 1 )^e na redução das taxas de rejeição de órgãos.^( 14 )^Além disso, a infusão de catecolaminas, como a noradrenalina, pode ser ajustada, com a finalidade de manutenção da pressão arterial média acima de 65mmHg, e viabilização dos órgãos para doação.^( 30 , 35 )^No entanto, em casos de choque misto neurogênico e hipovolêmico, é indicada a infusão da dopamina até 10µg/kg/minuto, evitando-se a dobutamina com o aumento do consumo de oxigênio e a noradrenalina, pois provoca-se vasoconstrição,^( 36 )^exigindo, assim, do profissional de saúde uma avaliação detalhada, no intuito de identificar as causas básicas e determinar as condutas assertivas.

A administração de corticosteroides, como hidrocortisona, potencializa também a estabilidade hemodinâmica e pode reduzir o volume acumulado em casos de ME com insuficiência circulatória, já que atua como um bloqueador adrenérgico em pacientes com alguma deficiência adrenal.^( 19 , 21 )^Já a metilprednisolona é recomendada por sua ação anti-inflamatória em enxerto hepático, associada à redução inflamatória hepática pós-transplante.^( 12 )^Estudo comparativo entre corticoides de alta dose (metilprednisolona) e baixa dose (hidrocortisona) identificou que a utilização de corticosteroide de dose baixa não resultou em piora do quadro pulmonar ou cardíaco, com órgãos transplantados comparados ao uso tradicional de metilprednisolona.^( 11 )^

A utilização de corticosteroides é baseada em achados comuns, e sua combinação com drogas vasoativas resultou em melhora significativa na aquisição de órgãos.^( 11 , 34 )^Estudo multicêntrico prospectivo controlado apontou que a administração de baixa dose de esteroides proporciona o desmame de vasopressores, e também diminui em mais de 20% a quantidade de vasopressores necessários para controlarem a insuficiência circulatória, permitindo redução significativa na necessidade de suporte inotrópico,^( 21 )^o que reforça a utilização do fármaco como aliado ao manejo hemodinâmico na ME.

Em relação aos cuidados e controles imprescindíveis à manutenção do potencial doador, um protocolo de diretrizes para os cuidados considera ações como limites da temperatura corporal, limites de pressão arterial e controle de marcadores bioquímicos essenciais. De acordo com o protocolo, a ME paralisa funções termorreguladoras hipotalâmicas, levando a uma hipotermia progressiva, o que demonstra a importância da regulação da temperatura corporal, já que proporciona a homeostase térmica. Recomenda-se manter a temperatura corporal acima de 35^o^C (ideal entre 36C^o^e 37,5C^o^), com uso de cobertores térmicos, ventilador mecânico e infusão de fluidos intravenosos aquecidos.^( 12 )^

O principal desafio do manejo dos potenciais doadores é a instabilidade hemodinâmica, o que remonta à necessidade de um conhecimento abrangente acerca dos processos orgânicos que envolvem a ME, e das condutas indicadas para mitigação de tais alterações. Isso se dá pelo fato de muitos pacientes apresentarem hipoperfusão prolongada, ou seja, após a ME ocorre hiperatividade adrenérgica, o que leva à taquicardia, ao aumento da resistência vascular sistêmica, à elevação do consumo de oxigênio miocárdico e ao reflexo sistólico da hipertensão arterial.^( 12 )^

O primeiro evento, a hipoperfusão prolongada, classificada pelo autor como “tempestade simpática”, dura de 20 a 30 minutos, e é seguido por hipotensão arterial. Posteriormente, a segunda fase, caracterizada como “tempestade adrenérgica”, produz hipertensão arterial, provocando hipoperfusão decorrente da vasoconstrição transitória.^( 12 )^A monitorização hemodinâmica não invasiva para mensuração de débito cardíaco e perfusão tecidual permite a identificação dessas fases,^( 27 )^e assim, a intervenção precoce, com o objetivo de preservar os órgãos.

Existem diferenças significativas em relação à monitorização invasiva e à não invasiva, principalmente quando ocorre vasoconstrição exacerbada. Nesse caso, recomendam-se medidas invasivas para manutenção da pressão arterial, uma vez que são mais seguras e fáceis para a coleta de exames, por meio da gasometria arterial, bem como da determinação da pressão arterial.^( 12 )^Além disso, devido à instabilidade hemodinâmica e por possíveis complicações, a ECMO pode ser usada como estratégia para prevenir a parada cardíaca e preservar órgãos.^( 16 )^

Cuidados poucos citados, como o início ou manutenção da dieta enteral e a proteção ocular com gaze úmida, podem ser justificados pela maioria dos artigos abordarem questões medicamentosas e outros cuidados específicos para a estabilidade hemodinâmica. No entanto, o manual recomenda manter dieta para suprir 15% a 30% das necessidades diárias, mas suspender sua infusão se houver elevação das doses de drogas vasoativas e sinais de hipoperfusão tecidual,^( 1 )^constituindo este um cuidado primordial para manutenção da nutrição tecidual.

Embora a busca tenha sido limitada aos artigos com acesso livre nas bases de dados e por meio dos descritores listados, observou-se que assegurar a manutenção hemodinâmica na ME é essencial para a viabilidade do transplante. Para isso, é preciso que a equipe de profissionais da saúde que participam desse processo tenha conhecimento acerca das condições clínicas e de todos os aspectos que envolvem os cuidados e as condutas sobre a ME. Para tanto, seguir e sempre atualizar os protocolos baseados em achados na literatura são de suma importância para melhores desfechos sobre essa temática.

## CONCLUSÃO

As principais estratégias para a manutenção hemodinâmica do potencial doador de órgãos se baseiam em controles de parâmetros cardiovasculares e respiratórios, bem como em intervenções voltadas ao uso de fármacos.
